# Mandated or Voluntary Treatment of Men Who Committed Child Sexual Abuse: Is There a Difference?

**DOI:** 10.3389/fpsyt.2021.708210

**Published:** 2021-09-30

**Authors:** Fritjof von Franqué, Peer Briken

**Affiliations:** Institute for Sex Research, Sexual Medicine and Forensic Psychiatry, University Medical Center Hamburg-Eppendorf, Hamburg, Germany

**Keywords:** prevention, sexual violence, pedophilia, risk, need, responsivity, sexual offense, rehabilitation

## Abstract

Child sexual abuse is associated with multiple and often severe consequences for people who are affected by it. From the perspective of indicative prevention, the treatment of people who have sexually abused children represents one important strategy, with the assumption that there is often a risk for sexual recidivism. However, there is still very limited knowledge about how men who have not been convicted of child sexual abuse but participate in voluntary treatment (here called non-forensic clients) differ from those who have been convicted and undergo mandated treatment (here called forensic clients). This study compared 22 forensic and 22 non-forensic clients regarding pedophilic interests, static and dynamic risk factors, responsivity features, and treatment progress during an individualized treatment based on the principles of risk, need, and responsivity. We found neither differences in the rates in the DSM-5 diagnosis of pedophilic disorder, nor in risk and responsivity associated scores at the beginning of treatment. In both groups, a low to moderate risk for sexual re-offending was estimated. Both groups improved their functioning on dynamic risk and responsivity factors under treatment, while age at the beginning of therapy also had a positive effect on all outcomes. Non-forensic clients had a higher amount of responsivity associated resources than forensic clients during treatment. The limitations of these results and their implications for further research and prevention approaches are discussed.

## Introduction

Experiencingchild sexual abuse can be an antecedent for severe negative outcomes: Dworkin et al. (1) (p. 65) found in their meta-analysis with 238,623 individuals that “people who have been sexually assaulted report significantly worse psychopathology than un-assaulted comparisons (average Hedges' g = 0.61).” In addition, Cashmore and Shackel (2) summarized several studies that showed associations with physical impairments (e.g., injuries or chronic sensations of pain), psychological and behavioral problems (e.g., emotional dysregulation, sexual risk behavior, suicidal tendency) and intimacy deficits (e.g., problems with engaging in relationships). Finally, different researchers discussed a link between experienced sexual abuse and sexual offending in adolescence or adult life (3–6). For example, Jespersen et al. (7) compared 1,037 individuals with sex offenses to 1,762 participants with non-sex offenses in their meta-analysis. The authors found a higher prevalence of sexual abuse history among individuals with sexual delinquency (Odds Ratio = 3.36). Considering these results, it is obvious that mental health professionals should prevent child sexual abuse underall circumstances (8). To achieve this task, the development and implementation of interventions at a universal, selective and indicative level are necessary, analogous to their conception in the field of mental health (8–10).

The following study focuses on the treatment of persons, who have sexually abused children in their past, as one form of indicative prevention. A first vulnerable group in this area with a certain risk for sexual abuse might be individuals who have been convicted of such an offense. We refer to this group as forensic clients. Although the effectiveness of psychotherapy with this group has been challenged (11–15), treatment might be most effective if it follows the principles of risk, need and responsivity (RNR) (16, 17). According to Hanson et al. (17) these principles “should be a major consideration in the design and implementation of treatment programs for sexual offenders” (17) (p. 865). Consequently, different treatment providers around the world have been using them for their conception of sexual-forensic psychotherapy (16, 18–20). The RNR-principles can be defined as follows (21):

- The risk principle states that the higher the risk level is to which a person has been ascribed, the more treatment resources (like time in treatment and number of therapy sessions) the person should receive. The risk principle also follows the assumption that it is possible to assess the risk for a sexual recidivism (21). According to many authors, static and dynamic factors should be included in comprehensive risk assessments (21–24). Static risk factors cover an individual's past, remain relatively stable over time, and reflect the persistent tendency for recidivism. In contrast, dynamic risk factors cover the current situation, reflect the given potential for recidivism, and might change during any form of intervention (25).
- The need principle contains the directive that intervention should target dynamic risk factors or criminogenic needs. For example, attitudes supporting child sexual abuse or pedophilic interest are considered to be dynamic risk factors (16).
- Finally, the responsivity principle says that therapists maximize the chance for treatment success by tailoring the interventions to the special characteristics of their clients. There are two components of this principle: General responsivity implies that programs for individuals with delinquency should be based on the cognitive-behavioral paradigm. Specific responsivity refers to personal characteristics of clients that should lead to a different mode and style of therapy, implying different forms of treatment for different subgroups of clients.

Although the treatment of forensic clients seems reasonable for indicative prevention, researchers and treatment providers pointed to the possibility that another vulnerable subgroup with a risk for committing child sexual abuse might exist. A meta-analysis of Stoltenborgh et al. (26) with 9.000.000 participants illustrates this assumption. In this analysis, the prevalence of child sexual abuse in self-report studies was 12.7%, while a prevalence rate of only 0.4% was found in so-called informant studies (i.e., “reports of professionals, dossier or chart reviews, and informant observations of children such as teachers observing their students in primary schools,” p. 80). Besides methodological issues, this result might reflect that some individuals, who committed child sexual abuse and therefore have a certain risk for sexual recidivism, were never reported to the authorities and–subsequently–would never have had access to treatment forms associated with the criminal justice system. We refer to this subgroup as non-forensic clients. From the perspective of indicative prevention, it might be crucial to conceptualize what motivates and characterizes these individuals, how they could be reached sufficiently, and how effective programs for this subgroup might look. Beier et al. (27) suggested that pedophilic interest is highly likely in these individuals, a public media campaign problematizing this pedophilic interest might be suitable to reach this subgroup efficiently and that a treatment approach should focus on associated problems and behavioral control of pedophilic interest. As a consequence, the Prevention Project Dunkelfeld (PPD) was founded in Berlin in the year of 2005. A special media campaign was designed and a treatment program was implemented. The first results regarding the therapeutic effects were published in 2015. After 10 years of implementation, the authors conclude that a relevant subgroup for indicative prevention could be reached and treated and that their program could reduce dynamic risk factors (27) and therefore effectively contributes to the prevention of child sexual abuse. However, the assumption, evaluation, and conclusion made by Beier et al. (27) have been criticized in terms of effectiveness and efficiency (28–31).

In the last few years, there has been a growing interest in the difference between forensic and non-forensic clients [e.g., (32–34)]. However, to the authors' best knowledge, not a single publication can be found that directly discusses differences between both groups being in treatment. Therefore, based on the background and the criticism given above, the purpose of our exploratory study was to gain knowledge regarding the following areas:

1. It has been assumed that non-forensic clients have more often a pedophilic interest compared to forensic clients (27).
2. It is unclear whether the risk level of non-forensic clients is lower than the risk level of forensic clients.
3. Since RNR-based treatment seems most promising with forensic clients (16, 17), it seems reasonable that this approach should also be successful with non-forensic-clients. However, we assume that such treatment might even be more fruitful with non-forensic clients since they could have a stronger motivation for therapy: Usually, forensic clients are legally obliged to treatment by different authorities. This should lead to a higher amount of perceived coercion and reactance since the request for treatment might be experienced as a loss of free choice (35). From this perspective, resistance against therapy can be interpreted as a reaction to restore one's freedom. Therapists might deal with such clients using reactance-matched interventions [i.e., emphasis on personal autonomy; (36)]. However, it should take more time in therapy to reduce reactance and to develop an intrinsic motivation to change.

In sum, the aim of this study is to compare convicted and non-convicted clients who have sexually abused children regarding pedophilic interests, static and dynamic risk factors, responsivity features, and treatment progress during an individualized treatment based on the principles of risk, need, and responsivity.

## Method

### Background

The Hamburg Institute for Sex Research, Sexual Medicine, and Forensic Psychiatry has been offering outpatient treatment for people with paraphilic interests or sexual delinquency continuously since its foundation in 1959 (37). However, until 2011 treatment of clients with interest in minors not under mandated treatment was paid for by the health insurance companies. Therefore, personalized data (like name and diagnosis) had to be transferred, which was a critical circumstance for some clients. As a result, the institutes' prevention outpatient treatment center has been participating in the nationwide network *Do not become an offender* since 2011. This network connects 11 different institutions meeting certain quality standards. All institutions of the network offer treatment for persons with a sexual interest in minors and, inter alia, are at risk for committing child sexual abuse. Included in the treatment program are persons, (a) who have not yet committed child sexual abuse or consumed sexual abuse imagery, but fear to do so in the future, (b) who have committed child sexual abuse or consumed sexual abuse imagery, but are not (yet) known to the authorities, (c) who have committed child sexual abuse or consumed sexual abuse imagery in their past and have been reported or convicted, but all legal matters (e.g., court proceedings, supervision) have been completed. A diagnosis of pedophilia was not a prerequisite for admission at the time of inclusion for participation in this study. The network operates within the specific legal framework of German legislation (38): Therapists must respect medical confidentiality and are not allowed to report about clients who have sexually abused children in the past and are not at acute risk of becoming sexually abusive in the present. Further information can be found on the network's homepage (https://www.dont-offend.org/).

### Measures

#### Diagnostic Procedure

All subjects included in the study were checked for the DSM-5 criteria for pedophilic disorder due to research purposes. Other mental health problems were assessed using ICD-10, as is requested in German health care settings. The diagnosis was made only by therapists who had started or completed psychotherapeutic training and additional sex therapy qualifications. Besides a semi-structured clinical interview, diagnoses were based on the client's answers in different questionnaires, which are not included in the study presented here.

#### Evaluation of Static Risk Factors

##### STATIC-99

The STATIC-99 is an internationally widely used instrument for assessing the static risk for a sexually motivated reconviction in adult males, who had been charged or convicted for a sexual offense (39). The instrument was extensively revised in the year 2003 (40). This version is used in the present study. Another revision (Static-99R) includes a stronger weighting of age variables (41). However, given recent results of studies using the German-language version, the use of the original version rather than the revised version was recommended (42).

The measure utilizes relatively unchangeable static risk factors. These factors relate to the current age, the relationship and criminal history of the person being assessed, the given offense and the scope of violence as well as different characteristics of the victim of the sexually motivated crime. The STATIC-99 includes 10 items, all of which 9 items must be coded with 0 or 1. A score of one is given for an answer which is empirically associated with a new reconviction. In addition, one item (a prior sex offenses) is more strongly emphasized: according to the number of prior charges or convictions, a score between 0 and 3 can be given. Consequently, a total score between 0 and 12 might result in the measure. Many studies have provided information on the psychometric properties of the STATIC-99, including its predictive validity. According to different interpretation guidelines, interrater reliability was acceptable (>0.75 in nearly all studies), while the predictive validity was moderate to high (39, 43–45).

##### STATIC-C

The STATIC-C (19) is a custom-made measure for determining the static risk in persons who have sexually abused children in the past or are at risk. The objective of the instrument is to help therapists allocate therapeutic resources according to the risk principle during their treatment planning, but without having case information from multiple sources (like a criminal register)^1^. The STATIC-C was formulated analogously to the STATIC-99 (40), which justifies the naming of the procedure^2^. It contains 12 items, of which nine items must be coded with 0 or 1. Two items must be coded with 0, 1, or 2 and one item must be coded with 0, 1, 2, or 3. The items contain the client's age (1 = older than 25 years), relationship history (1 = never in a relationship of 2 years), non-sexual violence (1 = at least one reported incident of actual, attempted, or threatened harm to another person), reported prior convictions, DSM5-diagnosis of pedophilic disorder (1 = pedophilic disorder, non-exclusive type, 2 = pedophilic disorder, exclusive type), ICD-10-diagnosis of other paraphilic disorder (1 = paraphilic disorder except sadistic disorder, 2 = sadistic disorder), diagnosis of personality disorder as well as number (1 = two different persons, 2 = three different persons, 3 = four or more different persons) and sex (1 = male) of a person affected plus their relationship with the client (1 = strangers to each other; 1 = unrelated). Therefore, a total score between 0 and 16 might result in the measure. Scores higher than five points reflect a high-risk level. We investigated the reliability and validity of the measure in a pre-test study (46): Two investigators independently coded 17 randomly selected cases from 80 different files of forensic clients. Interrater reliability for the 12 items resulted between 0.56 and 1.0 (Cohen's Kappa), while the reliability of the total score was 0.96 (ICC1). In terms of predictive validity, an AUC-value of 0.74 resulted in a retrospective study of 80 forensic cases. However, this value did not reach statistical significance due to the small number of sexual reconvictions (3.8%).

#### Evaluation of Dynamic Risk and Responsivity-Factors

##### Therapist Rating Scale (TRS-2)

The Therapist Rating Scale [TRS-2; (47) for the German translation (48)] is a 10-item measure for the evaluation of treatment success in sexual-forensic psychotherapy concerning dynamic risk and engagement factors. The measure contains the items Sense of Agency, General Empathy, Prosocial Attitudes, Adequate Coping Skills/Styles, Adequate Intimacy Skills, Positive Self-esteem, Good General Self-Regulation, Good Sexual Self-Regulation, Understanding Risk-Factors and Quality of Future Life Plans. For each item, three to five descriptors specify the content of the item. For example, the descriptors of the item Good Sexual Self-Regulation are

- doesn't use sex to cope,
- is not preoccupied with sex,
- has normative sexual interests, and
- has a healthy approach to sexuality.

Coming from a strength-based approach, the authors formulated all items as current functioning on dynamic risk and responsivity factors. Therefore, treatment providers might consider the items as targets of sexual-forensic treatment.

Items are scored along the dimensions (1) intellectual understanding and (2) acceptance/demonstration on a four-point scale, with higher scores indicating normative functioning in the skills and abilities necessary for desistance from sexual offending:

1. = unsatisfactory, needs to redo treatment component;
2. = approaching normative functioning, starting to understand and see value in topic/category, may achieve level 3 post-treatment;
3. = normative functioning, average functioning, mostly achieves target of treatment, might still have a little work to do, but no worse than non-offenders;
4. = optimal functioning, significantly better than average, most group participants will not achieve this level on any topic or category.

As a consequence, a total score in the measure varies between 20 and 80. As a heuristic, a score >50 may be indicative of treatment success, whereas scores <45 point to a demand for (further) treatment.

Comparing the items of the TRS-2 with descriptions of dynamic risk factors for sexual recidivism, it becomes clear that only the items Prosocial Attitudes, Adequate Coping Skills/Styles, Adequate Intimacy Skills, Good General Self-Regulation and Good Sexual Self-Regulation can be considered as associated with dynamic risk. According to the description in the manual, the remaining five factors could be considered as responsivity factors. Therefore, we summed up the items Prosocial Attitudes, Adequate Coping Skills/Styles, Adequate Intimacy Skills, Good General Self-Regulation and Good Sexual Self-Regulation to a sub-score reflecting a compensation of dynamic risk factors, while the sum of the items Sense of Agency, General Empathy, Positive Self-esteem, Understanding Risk-Factors and Quality of Future Life Plans contains the compensation of responsivity issues. Scores of these theoretically constructed subscales result in between 10 and 40 points.

The reliability of the TRS-2 in terms of intraclass correlation (ICC1) was 0.90 for the dimension intellectual understanding, 0.96 for the dimension demonstration, and 0.95 for the total score of the scale (47). To ensure that the measure is done consistently across raters, we did a pre-test with most therapists included in the given study (49). According to our findings, the reliability (ICC1) of the TRS-2 was 0.89 for the dimension intellectual understanding, between 0.88 for the dimension demonstration, and 0.90 for the total score.

Predictive validity of the measure was demonstrated in a retrospective study with 96 participants with sexual delinquency (of whom 21 individuals sexually reoffended). During a follow-up period of 5.84 years (SD = 3.60), an AUC = 0.77 resulted in the total score while the analysis yielded an AUC = 0.77 for the dimension intellectual understanding and an AUC = 0.77 for the dimension acceptance/demonstration (47).

##### STABLE 2007

The Stable-2007 (50) was designed to measure dynamic risk for sexual reoffending against children or adults. The instrument includes 13 dynamic risk factors that are divided into the domains (1) significant social influences (one item), (2) intimacy deficits (two items), (3) general self-regulation (four items), (4) sexual self-regulation (three items) and (5) cooperation with supervision (one item). All items are assessed on a three-point rating scale. Accordingly, the total sum score of the measure can vary between 0 and 26 points and be assigned to one of three categories: low (0–3), moderate (4–11), and high (>11). In combination with the Static-99, an actuarial overall risk rating with 5 risk levels is available.

Previous studies found satisfactory to good interrater reliabilities for the procedure (51–53). According to a meta-analysis, the Stable-2007 total score and risk categories are a good predictor of sexual recidivism (54). When used in combination with the Static 99, the Stable-2007 showed incremental validity in predicting sexual recidivism beyond the static factors.

### Participants

**Figure 1:**
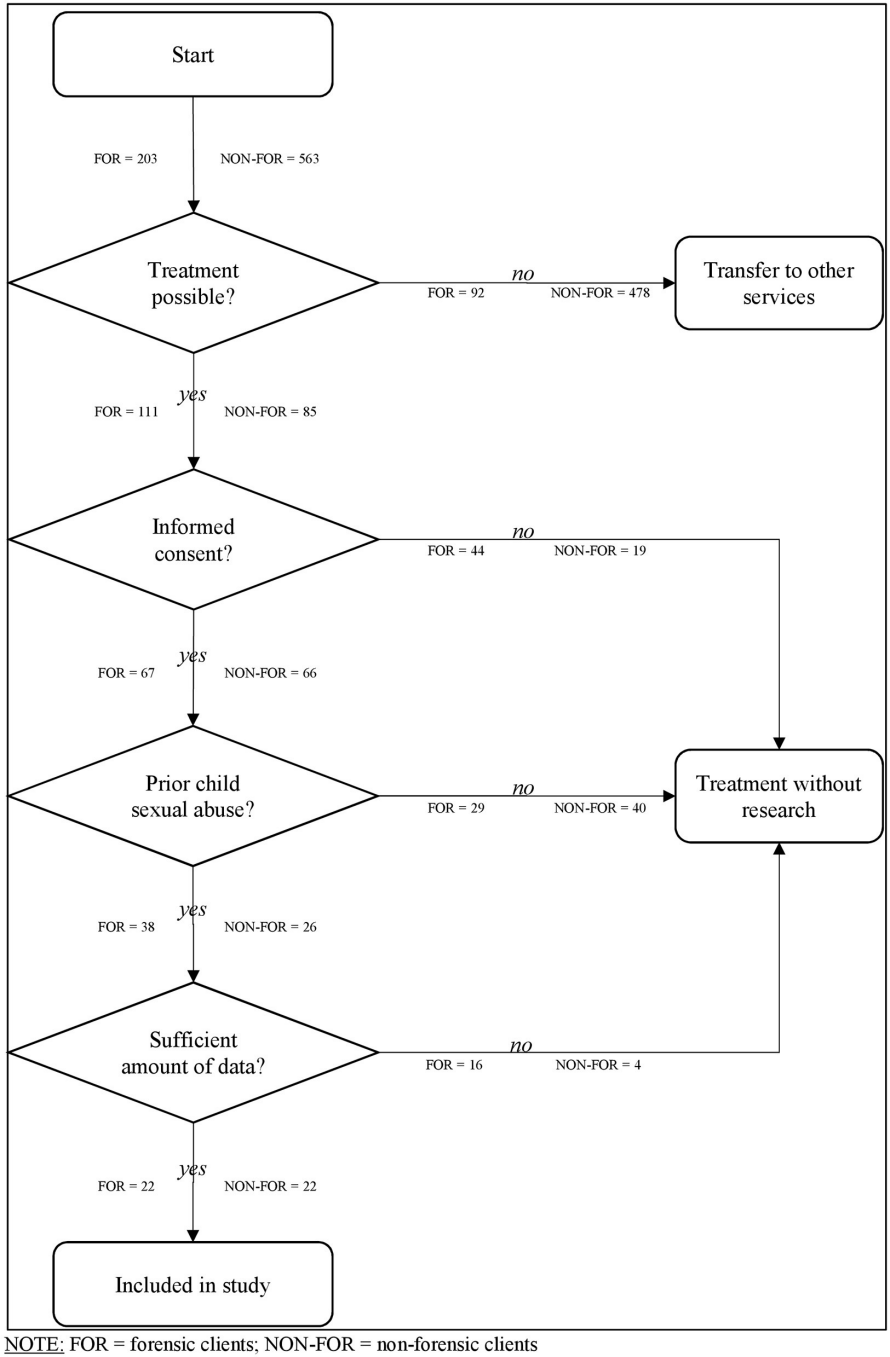
Inclusionprocess of the study. FOR, forensic clients; NON-FOR, non-forensic clients.

Selection criteria for inclusion of study participants are listed in Figure 1.

Forensic clients: Between April, the 1st of 2012, and July, the 1st of 2017, 203 individuals had been referred to the Institute by court or prisons to evaluate treatment indication. Of these, 92 individuals did not fulfill inclusion criteria for the program. Reasons were too long travel distance, hands-off offenses only (the program receives no payment for these clients), young age (between 18 and 23 years), acute substance problems, psychotic or obsessive-compulsive symptoms, acute suicidal thoughts, and impulses. Predominantly, individuals with child pornography offenses only or acute substance use problems were excluded. 111 persons were admitted to treatment, of whom 67 (60%) gave their informed consent that their data may be used for scientific purposes. Of these, 38 clients sexually abused a child in their past and thus were included. During data entry, 16 persons had to be excluded because of missing data. We examined the risk level to detect a potential selection bias. According to the exact Mann and Whitney *U*-Test (*U* = 167.5, *z* −0.25, *p* = 0.80, *r* = −0.25), there was no significant difference between selected participants (*n* = 22, M = 2.41, Mdn = 19.11) and non-selected participants (*n* = 16, M = 2.50, Mdn = 20.03) in the STATIC-99 total score. In the end, 22 forensic clients could be included in this study.

Non-forensic clients: Between April, the 1st of 2012, and July, the 1st of 2017, 563 interested parties sought contact with the project's office using telephone calls or E-Mails. Only 262 of these persons resided in Hamburg/ Germany and could thus be considered for treatment due to the city's funding of the project. In addition, 115 persons did not report their residence. These persons used an E-mail for the first contact, but did not accept our request for a further telephone call. In 65 cases, child sexual abuse or the consumption of child sexual abuse imagery was reported to authorities, but the legal proceedings had not been completed. Also, the legal status of 17 cases remained unclear since these persons used an E-mail for the first contact but did not accept our request for a further telephone call. Of the remaining 180 persons, only 169 individuals took part in a first consultation. Of these, 84 participants did not fulfill inclusion criteria for the program. Reasons were young age (between 18 and 23 years), acute substance problems, psychotic or obsessive-compulsive symptoms (related to being pedophilic), acute suicidal thoughts, and impulses. Predominantly, individuals with substance use-problems or with obsessive-compulsive disorder who thought they had pedophilic interest were excluded. At the end, 85 individuals started the course of treatment. Only 66 participants (78%) gave their informed consent that their data might be used for scientific purposes. Of these, 26 clients sexually abused a child in their past and were included in the analysis, since the focus of the current study was sexual abuse. During data entry, additional four participants had to be excluded because of missing data. In the end, 22 non-forensic clients from the prevention project were included for data analysis.

### Procedure

Both groups underwent an initial diagnostic process of 6–10 h and individualized sexual-forensic psychotherapy thereafter. During the diagnostic process, sexual history, static and dynamic risk factors for child sexual abuse, and responsivity-factors were assessed. In this study, we used the STATIC-C and the TRS-2 to estimate client's risk level. In addition, we also measured criminogenic needs and responsivity factors with the TRS-2. According to the RNR-Modell (16, 21) and the Structured Professional Judgement–Approach [SPJ; (25)], an individualized treatment plan was formulated at the end of the diagnostic process, containing the following aspects:

- Setting: In principle, the program offers group and individual treatment. Group therapy contains 90 min of group treatment as a so-called slow open group led by two group therapists weekly. Individual treatment sessions of 30 to 60 min occurred every one, two, or 4 weeks. Referral to group vs. individual treatment is adjusted to the individual's risk level and specific responsivity factors. In general, clients receive individual treatment or group therapy. Only in some cases, clients started with group therapy and changed to individual therapy (or vice versa) due to acute crisis, unmet dynamic risk factors, or unseen responsivity issues.
- Treatment of specific responsivity factors: The three most relevant responsivity-features were taken into account. The aim was to increase treatment engagement as a necessary condition for changing the relevant dynamic risk factors in the initial phase. For example, a small amount of sense of agency might be enhanced using motivational interviewing.
- Treatment of relevant dynamic risk factors: An individualized delinquency hypothesis with the three most relevant dynamic risk factors was formulated (25), which explained an individual's sexually abusive behavior in the past. Moreover, for each relevant dynamic risk factor, a therapeutic approach and specific interventions or techniques were outlined. For example, child sexual abuse supportive beliefs could be challenged within a cognitive behavioral approach using role-play or within a psychodynamic approach using interpretations of transference/countertransference. Interventions from a specific approach were conducted only by therapists who were trained in that specific approach.

Based on the treatment plan, all participating therapists discussed the final allocation to further interventions in a case conference. Treatment was provided by eight clinical psychologists or physicians also specialized in sex therapy. Two therapists were trained in cognitive behavioral therapy, while six therapists conducted psychodynamic approaches. In addition, three therapists were also trained in Motivational Interviewing. Furthermore, three persons had taken courses in further therapeutic approaches, i.e., transference-focused therapy, clarification-oriented therapy, and crucible therapy. Also, psychiatric treatment or medication was available in addition to psychotherapy. Every year, the treating therapist evaluated treatment progress by re-assessing dynamic risk-factors and specific responsivity-features. If necessary, the treatment plan was re-formulated (19, 55). According to the nature of the treatment plan and the variety of therapeutic approaches in the team of therapists, the treatment of these clients was quite variable. We use the term individualized treatment to refer to this circumstance.

### Design

The present study mainly used a so-called static group comparison (56), within which two groups, which were not randomly selected, were compared with one another at the beginning of therapy in terms of their characteristics on diagnostic variables, static and dynamic risk factors and responsivity factors. The samples differ in terms of the discovery of the child sexual abuse they had committed (sentenced vs. not detected) and the reason for treatment (mandatory vs. voluntary).

Only for the evaluation of treatment success did we use a longitudinal design, comparing annual assessments of dynamic risk and responsivity factors during the course of therapy. Thereby the design was unbalanced, meaning that a different number of observation results of the participants.

### Statistics

We used Fisher's Exact Test for the examination of the relationship between the samples and other categorical variables. As effect size, we calculated odds ratios, if possible. Comparing the samples in ordinal scaled variables, we used the exact *U*-Test. As effect sizes, we compute *r* = Z/√N. In general, we used a α <0.05 significance level. In necessary cases, we corrected the alpha level according to the Bonferroni method.

For the investigation of differences in the groups during the course of treatment, we performed a so-called multilevel linear effect analysis. Multilevel linear models are capable of handling correlated data, which are common in repeated measurements. Besides, estimations of parameters using these models are robust in cases of unbalanced designs, meaning a different number of observations per participant. Finally, linear mixed models allow to investigate fixed and random effect components. Fixed effects are defined as variables with all conditions of interest included in the data. Typically, fixed effects are those variables whose influence on the outcome is of primary interest. Fixed effects are not restricted to a particular scale level of the included variables. Accordingly, both categorical and metric variables can be included in a model as fixed effects. In contrast, random effects are defined as variables if a random sample of all possible conditions exists in the given data. For instance, this might be a random selection of participants. Typically, random effects in a linear mixed model are those variables whose influence on the outcome should be controlled (57).

For all multilevel linear models, we used the TRS-2- total score as outcome, which was assessed annually by therapists as long as a participant was in treatment. Accordingly, this variable is considered time-varying. As fixed effects, we used (1) the number of years a participant was in treatment at the time of the respective TRS-2 rating, (2) treatment group (forensic vs. non-forensic clients) and (3) age at the beginning of therapy. While the number of years in treatment increased with the number of TRS-2 ratings and therefore must be considered time-varying, age at the beginning of therapy and treatment group are time-invariant factors that did not change during the course of therapy. Statistically, we wanted to control for the repeated measures as well as for subject-specific intercepts and slopes: We began the modeling process by including the TRS-2 total score and all fixed effects, while we statistically controlled for the repeated measurement (initial model). We assumed a first-order autoregressive covariance structure (called AR1) for the repeated measures, which is often considered by various authors to fit a longitudinal design (58). Subsequently, we included one additional random effect. For all random effect models, we assumed a variance components covariance structure (VC) in the first step. This approach allowed us to initially look at the variance of the random effects in isolation. For random intercepts and slopes models, however, we selected an unstructured covariance structure (UN) in a second step. This allowed us to examine the covariance of the random effects in more detail. This approach has been recommended by Field (58). Using the chi-square likelihood ratio-test, we compare two models by the difference of their −2 log-likelihood –values. If a model proved to be statistically significant in relation to the previous model, we used this model as a starting point for the inclusion of the next random effect. If we did not observe a significant effect, we returned to the initial model or to the last one that had shown a statistically better model fit. Lastly, we used the model with the best model fit to investigate change in dynamic risk and responsivity associated factors separately. Therefore, we changed the outcome of the final model twice: First, we used the risk associated scores of the TRS-2 as the outcome. Second, we used the responsivity associated scores of the TRS-2 as the outcome. Parameter estimation was based upon the maximum-likelihood-method. Looking for deviations from homoscedasticity and normality, we visually inspected the residual plots. For all statistical computations, we used IBM SPSS Version 24 for Windows.

## Results

### Descriptive Data

Table 1 shows an overview of the descriptive data in both sub-samples.

**Table 1:** Descriptivedata of forensic (*n* = 22) and non-forensic clients (*n* = 22).

	**Forensic**	**Non-forensic**
**Sociodemographic data**		
Mean age in years (SD)	44.45 (10.49)	39.23 (11.41)
Number of subjects with more than 10 years in school (% within group)	13 (59%)	17 (77%)
Number of subjects with a job (% within group)	14 (64%)	15 (68%)
Number of subjects with an intimate relationship (% within group)	6 (27%)	10 (45%)
Number of subjects with children (% within group)	8 (36%)	7 (32%)
Number living alone (% within group)	15 (68%)	10 (46%)
**Treatment history**		
Number of subjects with previous therapy without sexual therapy (% within group)	2 (9%)	13 (59%)
Number of subjects with previous sex therapy (% within group)	4 (18%)	10 (46%)
Number of subjects with treatment during detention (% within group)	14 (64%)	0 (0%)
**History of antisocial behavior**		
Number of subjects who committed child sexual abuse in the last 6 months (% within group)	22 (100%)	9 (41%)
Number of subjects who committed child sexual abuse in their lifetime prior the last 6 months (% within group)	7 (32%)	21 (96%)
Number of subjects with other antisocial behaviors in their lifetime (% within group)	8 (36.4%)	6 (27.3%)
**Current treatment**		
Mean treatment length in months (SD)	33.18 (11.01)	28.05 (13.08)
Treatment setting		
Number of subjects with individual therapy only (% within group)	11 (50%)	14 (64%)
Number of subjects with group therapy only (% within group)	7 (32%)	5 (23%)
Number of subjects with group and individual therapy (% within group)	4 (18%)	3 (14%)
**Current assessments**		
Mean time in months between admission and first assessment (SD)	3.55 (4.00)	4.32 (2.75)
Number of Assessments		
Two assessments and ~1 year in treatment (% within group)	2 (9%)	10 (46%)
Three assessments and ~2 years in treatment (% within group)	12 (55%)	4 (18%)
Four assessments and ~3 years in treatment (% within group)	6 (27%)	6 (27%)
Five assessments and ~4 years in treatment (% within group)	2 (9%)	0 (0%)
Six assessments and ~5 years in treatment (% within group)	0 (0%)	2 (9%)

### Frequency of Pedophilic Disorder

In the forensic sample, 16 persons (72%) met the criteria for the diagnosis of pedophilic disorders according to DSM-5 criteria, while in the non-forensic group a pedophilic disorder was diagnosed in 21 persons (91%). There was no significant association between the subsamples and the diagnosis of the pedophilic disorder in Fisher's exact Test [χ2_(1)_ = 4.25, *p* = 0.10]. According to the calculated odds ratio, the chance of being diagnosed with a pedophilic disorder was 7.88 (95% CI = 0.86, 72.12) times higher in the non-forensic group. For the analysis of the three main criteria and the six specifiers, we corrected the alpha level according to Bonferroni method to α_corr_ = α/9 = 0.006. According to Fisher's exact Test we found no significant differences between the subsamples. Table 2 contains the descriptive results of the frequency distributions in the forensic and the non-forensic group.

**Table 2:** Frequencies (%) of pedophilic disorder and diagnostic subcriteria in forensic (*n* = 22) and non-forensic clients (*n* = 22).

	**Forensic**	**Non-forensic**
Pedophilic Disorder according to the DSM-5	16 (73%)	21 (96%)
DSM-5 A-Criterion
Over a period of at least 6 months, recurrent, intense sexually arousing fantasies, sexual urges, or behaviors involving sexual activity with a prepubescent child or children (generally age 13 years or younger).	16 (73%)	21 (96%)
DSM-5 B- Criterion
The individual has acted on these sexual urges, or the sexual urges or fantasies cause marked distress or interpersonal difficulty.	17 (77%)	22 (100%)
DSM-5 C- Criterion
The individual is at least age 16 years and at least 5 years older than the child or children in Criterion A	17 (77%)	22 (100%)
Exclusive type	4 (18%)	4 (18%)
Non-exclusive type	11 (50%)	18 (82%)
Sexually attracted to males	6 (27%)	3 (14%)
Sexually attracted to females	9 (41%)	16 (73%)
Sexually attracted to both	0 (0%)	3 (14%)
Limited to incest	1 (5%)	0 (0%)

*Fisher's Exact Test was used for all comparisons between the subsamples. Regarding the pedophilic disorder, the significance level was set at α <0.05. For the analysis of the subcriteria and the specifiers, a Bonferroni corrected α_corr_ = α/9 = 0.006 was used. All comparisons were nonsignificant*.

### Static Risk Factors

**Figure 2:**
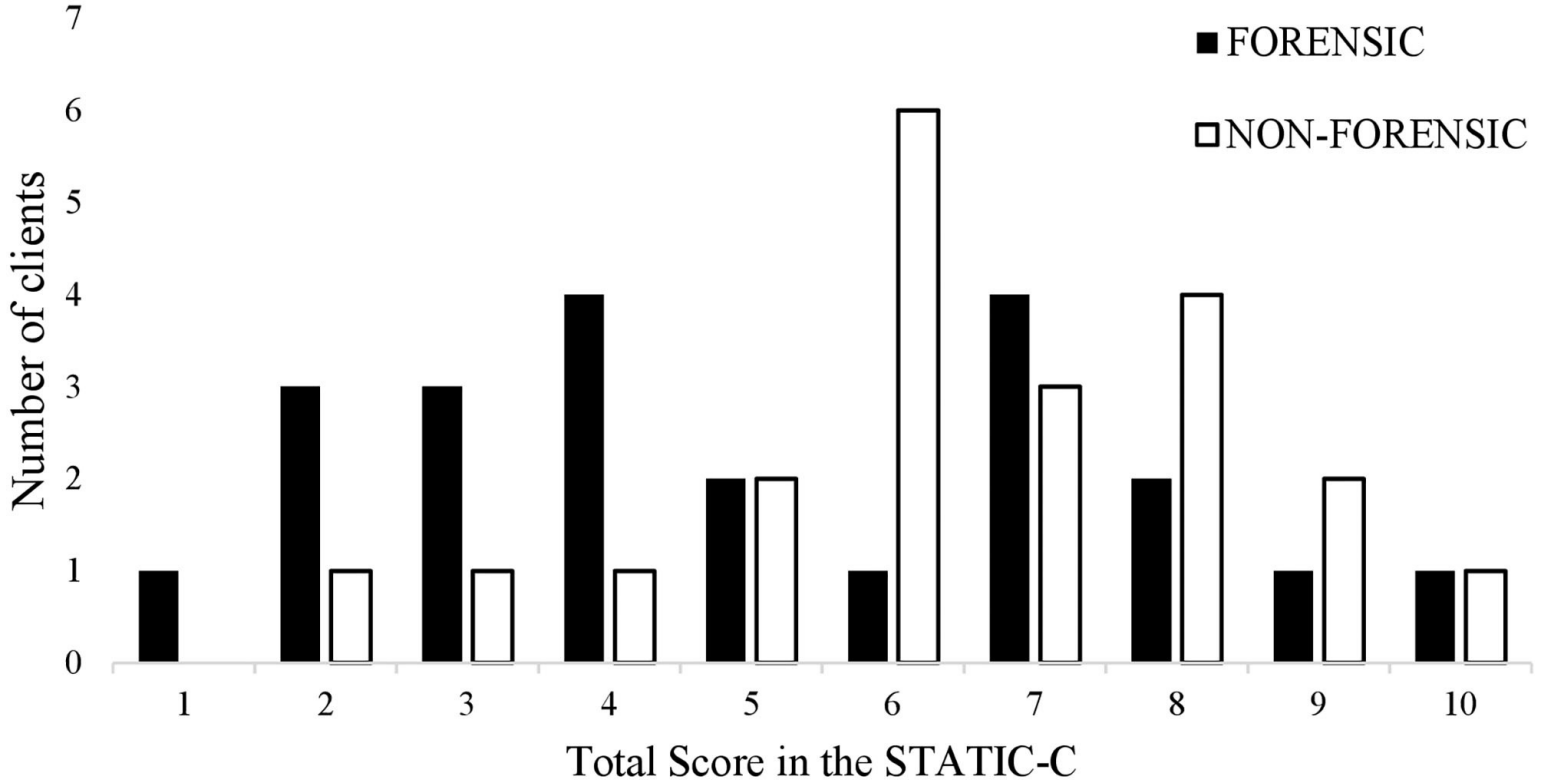
Number of forensic and non-forensic clients with a specific total score in the STATIC-C.

Figure 2shows the frequency distribution of sum scores in the STATIC-C for the different treatment groups.

According to the exact Mann and Whitney *U*-Test (*U* = 170.0, *z* = −1.703, *p* = 0.09, *r* = −0.25), levels of static risk did not differ significantly between forensic clients (M = 5.05, Mdn = 19.23) and non-forensic clients (M = 6.32, Mdn = 25.77).

Also, we used the STATIC-99 with the forensic subsample. The mean sum score of the measure was 2.45 (SD = 1.95, Mdn = 2.00). Concerning the interpretation guideline of the measure, the forensic subsample was rather a low to moderate risk group on average. Nine individuals (20.5%) of the forensic group were categorized as low-risk, whereas seven persons (15.9%) were included in the category low to moderate risk. Four participants (9.1%) were classified in the category moderate to high-risk. Only two persons (4.6%) fell in the high risk category. Overall, there was a significant association between the sum scores of the STATIC-C and the STATIC-99 (*r* = 0.58, *p* < 0.01).

### Dynamic Risk Factors

Table 3 gives an overview of risk-associated mean item scores in the TRS-2 at the beginning of treatment for the different subsamples.

**Table 3:** Mean scores of forensic (*n* = 22) and non-forensic clients (*n* = 22) in TRS-10- items associated with dynamic risk as well as corresponding effect sizes.

**TRS-2-Items**	**Forensic clients**		**Non-forensic clients**		**Effect size^a^**	
	**Understanding**	**Demonstration**	**Understanding**	**Demonstration**	**Understanding**	**Demonstration**
	**M (SD)**	**M (SD)**	**M (SD)**	**M (SD)**	**r**	**r**
Prosocial Attitudes	2.55 (0.67)	2.27 (0.70)	2.55 (0.80)	2.27 (0.83)	−0.01	−0.01
Adequate coping skills/styles	2.05 (0.65)	1.68 (0.48)	2.18 (0.66)	1.77 (0.61)	−0.11	−0.06
Adequate intimacy skills	1.95 (0.79)	1.64 (0.65)	1.91 (0.75)	1.73 (0.77)	−0.03	−0.05
Good general self-regulation	2.18 (0.59)	2.09 (0.61)	2.09 (0.68)	1.82 (0.66)	−0.06	−0.21
Good Sexual self-regulation	1.77 (0.61)	1.64 (0.58)	1.45 (0.67)	1.27 (0.46)	−0.28	−0.33
Total: functioning on dynamic risk factors	10.50 (2.35)	9.32 (2.19)	10.18 (2.67)	8.89 (2.15)	−0.09	−0.11

*Increasing scores reflect normative functioning and therefore are negatively associated with dynamic risk factors. The exact Mann und Whitney U-Test was used for all comparisons between the subsamples. Regarding the total score, the significance level was set at α <0.05. For the analysis of the single items, a Bonferroni corrected α_corr_ = α/5 = 0.01 was used. All comparisons were nonsignificant*.

a *The effect size r was calculated as Z statistic divided by the square root of the sample size (Z/√N)*.

With respect to the exact Mann and Whitney *U*-Test we found no significant difference in the risk-associated total score of the TRS-2 (*U* = 215.0, *z* = −0.638, *p* = 0.53, *r* = −0.10) between the forensic group (M = 19.82; Mdn = 23.73) and the non-forensic group (M = 19.05; Mdn = 21.27). Likewise, there were no significant differences at the item level.

Also, we analyzed STABLE-2007 ratings of 16 Persons of the forensic subsample. The mean sum score was 7.69 (SD = 4.11, Mdn = 8.00, missing = 6). Concerning the interpretation guideline of the measure, the forensic subsample had a moderate risk score on average. Consequently, three individuals (18.8%) of the subsample were categorized as low risk, 10 persons (62.5%) were assigned to the category moderate risk, and three participants (18.8%) were classified as high risk. The association between the sum scores of the STABLE-2007 and the risk-associated TRS-2 scores was *r* = −0.41 (*p* = 0.12).

Finally, we looked at the overall risk levels using the combination of the STATIC-99 and the STABLE 2007 results in the forensic clients. The mean sum score was 1.94 (SD = 1.24, Mdn = 1.50, missing = 6). Concerning the interpretation guideline, the forensic subsample had a low to moderate risk level on average, when static and dynamic risk factors are combined: eight individuals (50%) had a low risk, and four persons (25%) a low to moderate risk level, two participants (12.5%) had a score resulting in a moderate to high risk level, while one individual (6.3%) reached the high risk category. Finally, one participant (6.3%) was classified as very high risk.

### Responsivity Factors

Table 4 contains responsivity associated mean item scores in the TRS-2 at the beginning of treatment for different treatment groups.

**Table 4:** Mean scores of forensic (*n* = 22) and non-forensic clients (*n* = 22) in TRS-2- items associated with responsivity factors as well as corresponding effect sizes.

**TRS-2-Items**	**Forensic clients**		**Non-forensic clients**		**Effect size^a^**	
	**Understanding**	**Demonstration**	**Understanding**	**Demonstration**	**Understanding**	**Demonstration**
	**M (SD)**	**M (SD)**	**M (SD)**	**M (SD)**	**r**	**r**
Sense of agency	2.36 (0.65)	1.95 (0.72)	2.45 (0.67)	1.98 (0.77)	−0.11	−0.03
General empathy	2.00 (0.69)	1.77 (0.69)	2.18 (0.91)	1.77 (0.61)	−0.11	−0.13
Positive self-esteem	2.14 (0.89)	1.82 (0.80)	2.36 (0.49)	2.05 (0.58)	−0.10	−0.17
Understanding risk-factors	1.95 (0.72)	1.71 (0.56)	2.18 (0.73)	2.00 (0.61)	−0.16	−0.23
Quality of future life plans	2.27 (0.55)	2.23 (0.61)	2.45 (0.67)	2.23 (0.61)	−0.18	0.00
Total: Functioning on responsivity factors	10.73 (2.51)	9.41 (2.19)	11.64 (2.44)	10.14 (2.12)	−0.19	−0.18

*Increasing scores reflect normative functioning and therefore are negatively associated with dynamic risk factors. The exact Mann und Whitney U-Test was used for all comparisons between the subsamples. Regarding the total score, the significance level was set at α <0.05. For the analysis of the single items, a Bonferroni corrected α_corr_ = α/5 = 0.01 was used. All comparisons were nonsignificant*.

a *The effect size r was calculated as Z statistic divided by the square root of the sample size (Z/√N)*.

There was no significant difference between the forensic clients and the non-forensic clients in the TRS-2 items associated with responsivity factors. Equally, no significant differences resulted in the responsivity-associated total score of the TRS-2 between the forensic clients (M = 20.14; Mdn = 20.00) and the non-forensic clients (M = 21.77; Mdn = 25.00) at the beginning of treatment (*U* = 187.00, *z* = −1.30 *p* = 0.20, *r* = −0.20).

For the forensic subsample, the correlation between the sum scores of the STABLE-2007 and the responsivity associated TRS-2 scores was not significant (*r* = −0.41, *p* = 0.12).

### Treatment Evaluation

Table 5 summarizes the results of the modeling process, using a multilevel linear effect analysis.

**Table 5:** Estimates of multilevel linear analysis of the TRS-2 total scores based on treatment group, years in treatment and age at the beginning of therapy.

	**Model 1**	**Model 2**	**Model 3**	**Model 4**
**Intercept**	32.45 (3.77)^**^	32.46 (3.84)^**^	30.94 (3.81)^**^	31.25 (3.80)^**^
**Fixed effects**				
Treatment group	−2.11 (1.97)	−2.10 (2.00)	−2.46 (2.00)	−2.41 (1.99)
Years in treatment at the respective TRS-2 rating	2.41 (0.48)^**^	2.38 (0.44)^**^	2.27 (0.52)^**^	2.28 (0.54)^**^
Age at the beginning of treatment	0.21 (0.087)^**^	0.21 (0.09)^**^	0.25 (0.09)^**^	0.25 (0.09)^**^
**Repeated measures^a^**				
AR1, diagonal	55.478 (9.18)^**^	34.80 (18.79)	14.94 (6.59)^**^	13.35 (5.78)^**^
AR1, Rho	0.96 (0.01)^**^	0.93 (0.05)^**^	0.83 (0.13)^**^	0.81 (0.14)^**^
**Random effects^b^**				
Variance intercepts across subjects		20.41 (19.45)	31.28 (10.04)^**^	34.54 (11.24)^**^
Variance slopes across subjects			5.49 (3.42)	6.92 (4.18)
Covariance intercepts and slopes				−2.59 (4.76)
**−2 Log-Likelihood**	876.35	875.90	872.75	872.44
df	6	7	8	9
**Change (comparison model)**		0.45 (Model 1)	3.59 (Model 1)	3.91 (Model 1)
df change		1	2	3

*Values in parentheses are the standard errors for the associated parameter estimates*.

a *a first-order autoregressive covariance structure (AR1) was assumed for the repeated measures*.

b *For model 2 and 3, a variance components covariance structure (VC) was assumed, while an unstructured covariance structure (UN) was selected for model 4*.

* *p <0.05*,

** *p <0.01*.

In all models, the number of years in treatment at the corresponding TRS-2 rating and the age of clients at admission were found to be statistically significant, whereas there was no significant difference between treatment groups. According to the 2-likelihood test, there was no significantly improved model fit by including the random effects or by accounting for their covariance. For this reason, we selected the initial model as the final one.

For this model, we found a statistical association between TRS-2 total score and years in treatment at the respective TRS-2 rating [b = 2.41; 95% CI = 1.46, 3.35; F_(1, 133.86)_ = 25.43; *p* < 0.01], while there was no significant effect for the treatment group [b = −2.11; 95% CI = −6.07, 1.85; F_(1, 43.68)_ = 1.16, *p* = 0.29] which means that both groups showed a change under treatment in the same direction. Additionally, age at the beginning of therapy [b = 0.21; 95% CI = 0.04, 0.39; F_(1, 42.19)_ = 5.94, *p* < 0.05] was significant.

Using the risk associated scores of the TRS-2 as the outcome, there was a significant effect for years in treatment at the respective TRS-2 rating [b = 1.28; 95% CI = 77, 1.79; F_(1, 137.29)_ = 24.81, *p* < 0.01] and age at the beginning of therapy [b = 0.10; 95% CI = 0.00, 0.19; F_(1, 42.59)_ = 4.38, *p* < 0.05], whereas treatment group was not significant [b = 0.00; 95% CI = −0.2.10, 2.12; F_(1, 44.10)_ = 0.00, *p* = 0.99].

Using the responsivity associated scores of the TRS-2 as the outcome, there was a significant association with years in treatment at the respective TRS-2 rating [b = 0.00; 95% CI = 0.57, 1.69; F_(1, 137.53)_ = 16.1, *p* < 0.05] and age at the beginning of therapy [b = 0.12; 95% CI = 0.02, 0.21; F_(1, 43.70)_ = 6.17, *p* < 0.05]. Here, the factor treatment group [b = −2.18; 95% CI = −4.30, −0.06; F_(1, 45.18)_ = 4.28, *p* < 0.05) was significant. This means that non-forensic clients had a higher amount of responsivity associated resources than forensic clients over the course of treatment.

## Discussion

Results of this pilot study show that the differences between forensic clients and non-forensic clients seem rather marginal. Regarding the diagnosis of pedophilic disorder, we found no differences in the rates between both groups. Consistent with this finding, forensic and non-forensic clients seem relatively similar in terms of risk. Measured with the STATIC-C, there was no significant difference in static risk factors. Based on this, it can be concluded that the client's previous history was roughly equally affected from a risk perspective. The same was true for dynamic risk and responsivity factors. Using the TRS-2, we measured the abilities and skills to compensate for criminogenic needs and engagement deficits. We found no significant difference in both subscales. Likewise, there were no significant differences on the item level of the TRS-2. These findings should be interpreted in relation to another result of our study: We found a low to moderate risk level in the forensic sample measured with the STATIC-99 in combination with the STABLE 2007. In addition, there was a significant correlation between the STATIC-99 and the STATIC-C in the forensic clients and an association between the TRS-2 and the STABLE 2007, although this was not significant. Based on these findings, we, therefore, conclude that the prevention group has a low to moderate risk level on average.

Regarding treatment change, we found no difference between the treatment groups, but a significant effect of years in treatment. This means that both groups improved in relevant abilities and skills in the same way, resulting in a change of approximately two points per year in the total score. We conclude that in principle an individualized procedure following the RNR-principles and linking relevant dynamic risk factors with psychotherapeutic interventions of different approaches seems applicable to both investigated groups. Our findings slightly changed when we used risk and responsivity associated scores with our initial model. On average, the treatment led to a change of risk and responsivity associated scores of one point per year. Again, but to a smaller extent, there was a significant association between treatment change and years in treatment. Again, we conclude that both groups improved their functioning in both areas. However, there was also a significant difference between forensic clients and non-forensic clients in the responsivity associated score. Non-forensic clients had about two more points in this scale during their course of treatment. This result was neither observable in the dynamic risk associated score, nor in the initial assessment. According to this, non-forensic clients had a stronger sense of agency and a higher quality of future life plans, more positive self-esteem, and greater capacities in general empathy and understanding personal risk factors. Except for empathy, this might reflect a stronger treatment motivation in non-forensic clients. This interpretation seems reasonable since only forensic clients have contact with the different authorities requesting their treatment.

Furthermore, our results showed that age at the beginning of treatment was positively associated with a better functioning on dynamic risk and responsivity factors. Regarding dynamic risk, this finding is consistent with the results and interpretation of Hanson (59), who assumes an improvement in self-regulation during the transition from the twenties to the thirties in participants with sexual offenses, but not a decline in deviant sexual interest until the age of 50. Considering the mean age around 40 years and the high rate of pedophilic disorders in our subsamples, this interpretation seems compatible with our results. With regard to the responsivity score, our results show that younger age is associated with lower resources in terms of sense of agency, quality of future life plans, self-esteem, understanding personal risk factors and capacities in general empathy. As previously stated, this might indicate lower treatment motivation in younger subjects. For this reason, additional interventions to promote treatment motivation in participants with young age might be indicated.

There are several limitations to our study. First, the non-forensic sample is not representative of all persons that seek preventive treatment in the network *Do not become an offender*. Clients who did not give informed consent or were from other institutions, may have, on average, higher risk profiles. Second, both subsamples in the study were relatively small. It is, therefore, possible that non-significant differences between the investigated groups (e.g., the frequencies of pedophilic disorders) might differ with larger samples. Third, we assume that both subsamples have a low to moderate risk level. With respect to treatment effectiveness, it is possible that our treatment approach leads to other effects with other risk groups. Forth, some of the measures used in our study are not sufficiently validated. Especially the STATIC-C might overestimate the individual's risk level, though the scores of the measure correlated significantly with the STATIC-99. Nevertheless, half of the forensic sample has been diagnosed with a high-risk level measured with the STATIC-C, while only two clients meet this criterion using the STATIC-99. Therefore, a cut-off score of 6 might be too low for diagnosing a high-risk profile. Future studies using the STATIC-C should therefore consider adjusting the intervals that define the risk levels of the measure. For measuring dynamic risk factors, nowadays there are better-validated instruments available, like the Violence Risk Scale—Sex Offender Version (60). Fifth, we did not provide any evidence of whether the risk principle was met in our study. Theoretically, duration in treatment should covary with clients' risk level. However, for such a test, all or at least a large proportion of participants would need to have completed the treatment program. Since our treatment program was ongoing, the risk principle could not be tested in the present context. Furthermore, the correlation between treatment duration and the risk level of clients could be moderated by specific responsivity factors. Taking them into account could also lead to a high duration of therapy for clients with a moderate risk level. For example, treating an individual with a moderate risk level and a personality disorder might require additional time to build a therapeutic alliance before addressing dynamic risk factors. Sixth, the investigation of the responsivity principle can be regarded as insufficient since we exclusively examined very specific responsivity factors. Seventh, the extent to which the therapy concept described here was implemented, has not been investigated in the present work. This problem of treatment integrity is also a key limitation of several other relevant studies in the field [e.g., (61, 62)]. Therefore, it is difficult to say what exactly causes positive changes during the course of treatment. Theoretically, these changes might be maturing effects that occur independently from treatment. Such an effect could be tested in future studies using a wait-list control group without any form of therapy. An alternative explanation is that there might be indeed a treatment effect. However, this is less associated with the specific approach outlined above, but with general treatment factors, like goal consensus, empathy, therapeutic alliance, and therapist features (63). Therefore, even if one assumes a treatment effect, it is difficult to interpret what exactly causes therapeutic changes. Eighth, therapeutic success was based on the judgments of those therapists, who treated the participants in the study. It is conceivable that their judgments were distorted to reduce cognitive dissonance in therapies with no progress. However, according to the study of Tozdan et al. (64), the therapist's initial assessments and their judgment of treatment success had predictive validity. Therefore, a strong confounding effect is rather unlikely. Lastly, treatment success was not measured with a strict outcome, like any reconviction with child sexual abuse. This is a key limitation since the aim of treatment is the prevention of future child sexual abuse. For this reason, future studies should consider measuring sexual recidivism after therapy, even though this could only be done using self-reports after a certain follow-up period.

From our point of view, our results are nevertheless encouraging for future indicative prevention approaches. Our study suggests that an RNR-based, but individualized treatment approach might be effective with forensic clients as well as with non-forensic individuals. Particularly, we think that the results speak against fundamental changes in the program since the treatment leads to a reduction in dynamic risk. Nevertheless, the results underline how much time therapeutic changes might need. Regarding the group differences in the responsivity associated scores, we think that our finding does not indicate fundamental changes in the way treatment is delivered. In theory, the responsivity principle states that non-criminogenic features of clients should be addressed during treatment to increase the chance for treatment success. However, since both groups improved equally in the total score of the TRS-2 during the course of therapy and especially in the risk-associated scale, the responsivity principle–at least concerning the factors contained in the TRS-2–seems to be sufficiently fulfilled. We, therefore, think that a modification of the procedure is not necessary with regard to the treatment groups. Both groups seem to have rather high resources so that treatment change could occur. However, considering the effect of age at the beginning of therapy, it might be helpful to at least examine young client‘s treatment motivation more closely or even to strengthenit by additional interventions.

## Data Availability Statement

Theoriginal contributions generated for this study are included in the article/supplementary material, further inquiries can be directed to the corresponding author/s.

## Ethics Statement

The evaluation of forensic clients was approved by the Ethics Committee of the German Society of Psychology in September 2012 (ID: MR 03_2012 rev.). For the non-forensic clients, the study was approved by the Ethics Committee of the Chamber of psychotherapists Hamburg in April 2015 (ID: 02/2015-PTK-HH). The patients/participants provided their written informed consent to participate in this study.

## Author Contributions

FFand PB: conceptualization, methodology, analysis and interpretation of results, and writing-review & editing. FF: writing-original draft preparation. PB: supervision, project administration, and funding acquisition. All authors reviewed the results and approved the final version of the manuscript.

## Conflict of Interest

The authors declare thatthe research was conducted in the absence of any commercial or financial relationships that could be construed as a potential conflict of interest.

## Publisher's Note

All claims expressed in this article are solely those of the authors and do not necessarily represent those of their affiliated organizations, or those of the publisher, the editors and the reviewers. Any product that may be evaluated in this article, or claim that may be made by its manufacturer, is not guaranteed or endorsed by the publisher.

## Footnotes

^1^This is not to say that risk-assessments should generally rely on information from only one source. In principle, risk assessments should take into account information from multiple sources whenever this is possible. However, the practical problem for the study population was that common risk assessment procedures (like the STATIC 99) may not be used without official data of a person's criminal history. Due to the nature of the non-forensic clients described here, this critical information was not available. Thus there was a need for an instrument without the necessity of objective data.

^2^However, it is not intended to suggest that the measure is comparably predictive of risk to the STATIC-99.
