# Corrigendum: Mandated or voluntary treatment of men who committed child sexual abuse: Is there a difference?

**DOI:** 10.3389/fpsyt.2023.1145593

**Published:** 2023-02-07

**Authors:** Fritjof von Franqué, Peer Briken

**Affiliations:** Institute for Sex Research, Sexual Medicine and Forensic Psychiatry, University Medical Center Hamburg-Eppendorf, Hamburg, Germany

**Keywords:** prevention, sexual violence, pedophilia, risk, need, responsivity, sexual offense, rehabilitation

In the published article, there was an error in Table 3 as published. For the non-forensic group, the means and standard deviations were incorrectly reported as 2.55 (0.80) for Understanding and 2.27 (0.83) for Demonstration. The correct Table 3 with the corrected means and standard deviations for Understanding 10.18 (2.67) and Demonstration 8.89 (2.15) and its legend appear below.

**Table 3:** Mean scores of forensic (*n* = 22) and non-forensic clients (*n* = 22) in TRS-10- items associated with dynamic risk as well as corresponding effect sizes.

**TRS-2-Items**	**Forensic clients**		**Non-forensic clients**		**Effect size** ^**a**^	
	**Understanding**	**Demonstration**	**Understanding**	**Demonstration**	**Understanding**	**Demonstration**
	**M (SD)**	**M (SD)**	**M (SD)**	**M (SD)**	***r***	***r***
Prosocial attitudes	2.55 (0.67)	2.27 (0.70)	2.55 (0.80)	2.27 (0.83)	−0.01	−0.01
Adequate coping skills/styles	2.05 (0.65)	1.68 (0.48)	2.18 (0.66)	1.77 (0.61)	−0.11	−0.06
Adequate intimacy skills	1.95 (0.79)	1.64 (0.65)	1.91 (0.75)	1.73 (0.77)	−0.03	−0.05
Good general self-regulation	2.18 (0.59)	2.09 (0.61)	2.09 (0.68)	1.82 (0.66)	−0.06	−0.21
Good sexual self-regulation	1.77 (0.61)	1.64 (0.58)	1.45 (0.67)	1.27 (0.46)	−0.28	−0.33
Total: functioning on dynamic risk factors	10.50 (2.35)	9.32 (2.19)	10.18 (2.67)	8.89 (2.15)	−0.09	−0.11

Increasing scores reflect normative functioning and therefore are negatively associated with dynamic risk factors. The exact Mann und Whitney U-Test was used for all comparisons between the subsamples. Regarding the total score, the significance level was set at α < 0.05. For the analysis of the single items, a Bonferroni corrected α_corr_ = α/5 = 0.01 was used. All comparisons were nonsignificant. ^a^The effect size r was calculated as **Z** statistic divided by the square root of the sample size (Z/√N).

The authors apologize for this error and state that this does not change the scientific conclusions of the article in any way. The original article has been updated.

